# Identity processes and distress: a person-centered analysis of ecuadorian university students

**DOI:** 10.1080/28324765.2026.2626604

**Published:** 2026-02-04

**Authors:** Barbara M. Gfellner, Karin Bartoszuk, Jim Deal, Fernanda Cordero-Hermida, Ana I. Cordoba

**Affiliations:** a Brandon University, Brandon, Manitoba, Canada; b East Tennessee State University, Johnson, TN, USA; c North Dakota State University, Fargo, North Dakota, USA; d University of Cuenca, Cuenca, Ecuador; e University of Valencia, Valencia, Spain

**Keywords:** Identity processes, identity distress, latent profile analysis, psychological symptoms/CCAPS, psychosocial maturity

## Abstract

This study extended the identity development framework using latent profile analysis of identity processes on the Dimensions of Identity Scale (DIDS) with identity distress on the Identity Distress Scale (IDS), an affective indicator of difficulties with relevant identity issues. The objective was to investigate person-centered identity functioning typically obscured in variable-centered mean level research and how these profiles associate with traditional measures of identity, mental health, and psychosocial adjustment. Participants were 412 Ecuadorian university students (median_age_ = 21 years; 67% female). They completed online surveys that included the DIDS, IDS and measures of psychosocial development, functional well-being, and mental health. Consistent with profiles found in the Netherlands, United States, and other countries the findings supported a five-profile model that included Troubled Diffusion (20.4%), Carefree Diffusion (5.4%), Foreclosure (11.6%), Undifferentiated (40%), and Achievement (24.5%). As expected, profile differences on ego strengths and achieved identity status provided criterion validity. Predictive validity was indicated with adjustment to university, perceived stress, optimism, agency, and psychological symptoms. Findings underscore the relevance of identity profiles among young adults in Ecuador, emphasize the importance of culture in identity development, and provide directions for further research and supportive services in relation to students’ mental health and well-being.

## Introduction

Identity development is a primary psychosocial focus of youth (Erikson, 1968). It involves constructing a coherent sense of self that integrates the past with present and future expectations. The process is evolving and typically includes searching for and evaluating relevant options that precede incorporating personal choices into one’s self-conceptualisation. In conjunction with establishing a coherent personal synthesis, identity confusion is a separate but related aspect of identity development. It includes difficulties in identity construction that often result in a fragmented, disorganised, inconsistent sense of self (Erikson, 1968).

Over the years, Erikson’s theoretical framework has been refined and extended in conjunction with contemporary changes in the modern world (Luyckx et al., 2011; Schwartz et al., 2013; Waterman, 2015). As described below, the identity development paradigm was operationalized by Marcia (1966) and later elaborated into dual cycles of identity processes given complexities in the functional components (Crocetti et al., 2008; Luyckx et al., 2005, 2008a, 2008b). More recently empirically based identity status models were developed (Crocetti & Meeus, 2015; Schwartz et al., 2011). The Eriksonian conceptualisation has generated decades of research (Branje et al., 2021; Kroger & Marcia, 2011; Kroger et al., 2010) and remains steadfast as a foundation of personal well-being (Crocetti et al., 2016; Kroger & Marcia, 2022; Schwartz, 2005).

The current study investigated person-centred profiles of identity processes with the Dimensions of Identity Scales (DIDS; Luyckx et al., 2005, 2008a) among university students in Ecuador. Identity Distress (IDS; Berman et al., 2004, 2009) was included in the profile analysis as an affective component of identity development that indicates uncertainty, upset, and functional difficulties with relevant identity issues. In comparison, the DIDS focuses solely on the future domain. The objective of this study was to extend identity theory with the inclusion of affective identity domains in conjunction with identity processes among a sample of university students in an understudied country (Carreno et al., 2024).

### Identity development

The identity status paradigm (Marcia, 1966) operationalized Erikson’s framework in two dimensions: exploration—involves examining, evaluating, and deciphering among various personal options; and commitment—deciding, selecting, and incorporating one’s choices typically from the range of considered alternatives. These dimensions are cross-referenced into four identity statuses: Foreclosure (commitment without exploration); Moratorium (focused exploration); Achievement (commitment after a period of exploration); and Diffusion (absence of involvement in either exploration or commitment). The identity status paradigm has generated decades of research (see Kroger & Marcia, 2022; Kroger et al., 2010 for extensive reviews). Achievement is the most resilient identity status in terms of mental health and adaptive functioning. Foreclosure has many strengths given its anchor in commitment. Moratorium is characterised by considerable stress and disruption associated with the quest to attain appropriate personal integration in the process of identity deliberations. Alternatively, Diffusion is a lack of concern or disinterest in identity work as reflected in the poorest adjustment.

Contemporary social changes led to the construction of dual cycle models to account for complexities in the identity development process (Crocetti et al., 2016; Luyckx et al., 2005, 2011). The first cycle of the Dimensions of Identity Scale (Luyckx et al., 2008a, 2008b) is consistent with Marcia (1966). It includes Exploration in Breadth, in which a broad consideration and evaluation of possible alternatives is examined in a process of self-discovery. This is followed by Commitment Making, the selection and incorporation of personal choices into one’s self-conceptualisation. During the second cycle, Exploration in Depth is a closer examination of one’s choices that leads to either reaffirmation of one’s commitments, Identification with Commitment, or a return to examining a wider range of options that precedes the process of Commitment Making. Ruminative exploration is the maladaptive process that involves a lack of clarity, confusion, brooding, procrastination, or avoidance in dealing with identity issues (Luyckx et al., 2008a).

Recently, latent profile or cluster analysis has been used to examine within-person differences in identity development that may be obscured in the analysis of sample means (Crocetti & Meeus, 2015). This method provides a view of identity configurations that affords more detailed information on identity development beyond mean comparison. In combination, these approaches advance our understanding of identity development (Crocetti et al., 2016). See Branje et al. (2021) for a review of recent identity research.

Identity development occurs within and is influenced by cultural and social factors that reflect the way in which individuals select and explore identity issues. Greater focus on culture and context is emphasised in the study of identity in our global world (Galliher et al., 2017). Indeed, differences in identity profiles of young people in different countries are related to cultural and contextual influences (Hatano & Sugimura, 2017; Skhirtladze et al., 2016).

This study is a person-centred analysis of identity processes on the DIDS (Luyckx et al., 2008a, 2008b) with the inclusion of identity distress (Berman et al., 2004, 2009; Gfellner et al., 2025) to assess how affective identity issues are resolved in identity construction. The objectives were to extend the identity framework with the inclusion of identity distress and our understanding of identity construction with an understudied university sample in Ecuador (Carreno et al., 2024).

### Identity distress

Changes in contemporary societies have extended the age span for identity development, given the increased time required for education, professional training, entering the work force, and the age of marriage and parenthood. Emerging adulthood (EA; Arnett, 2016) defined as 18 to 29 years of age refers to the post-adolescent period before young people are expected to assume adult roles and responsibilities. It is a socio-cultural construction attributed to global changes in the revolutions of women, youth, sexuality, and technology (Arnett, 2007). As a result, young people of legal age tend to perceive themselves as being in-between the stages of adolescence and adulthood. This is a characteristic of EA along with identity exploration and commitment, instability, negativity, optimism, and self-focus (Arnett, 2007; Arnett & Mitra, 2018; Arnett et al., 2014). A certain amount of discomfort and uncertainty about identity issues is normal. However, some individuals become overwhelmed to the extent that identity distress is dysfunctional and interferes with normal functioning (Berman et al., 2004, 2009). Following the DSM taxonomy for identity impairment, these authors developed the Identity Distress Scale to measure the extent to which individuals experience extreme stress, uncertainty, confusion, and inability to deal with identity issues that seriously interfere with adjustment. Considerable research indicates the importance of identity distress in the mental health of community (Gfellner & Cordoba, 2020; Palmeroni et al., 2020) and clinical samples (Kamps & Berman, 2011; Papazova et al., 2018; Samuolis et al., 2015; Scott et al., 2014; Wiley & Berman, 2013). According to developmental psychopathology, identity distress falls along a continuum that extends from normal to severe disability (Berman & Montgomery, 2014; Kaufman et al., 2014).

### Identity processes and identity distress

In terms of identity processes, **i**dentity distress positively related to moratorium the identity status that involves active exploration without commitment, and inversely with commitment (Berman et al., 2004; Berman & Montgomery, 2014). More recent studies in Belgium (Palmeroni et al., 2020), Italy (Sica et al., 2014), and Canada (Gfellner & Cordoba, 2017) reported negative associations with the commitment variables, substantial negative associations with maladaptive exploration, and to a lesser extent, with adaptive exploration. Alternatively, identity distress and commitment were not related among Spanish students, and this was attributed to culture and context (Gfellner & Cordoba, 2017).

### The ecuadorian context

Ecuador is an upper middle-income country (World Bank, 2024) that has undergone substantial socio-economic and political alterations. As with other Latin American countries, it has experienced major changes in the last half-century that shape EA. These include increased rates of literacy and post-secondary education, modest declines in the age of first marriage, and an increased age of first birth (Carreno et al., 2024). Education is a national priority in Ecuador with incentives for tertiary achievement (Guijarro-Garvi et al., 2022).

According to Hofstede (2015) cultural dimensions, Ecuador ranks among the highest countries in the world for collectivism. This is reflected in strong norms and expectations that emphasise interpersonal relationships, group loyalty, cohesion, and identity, with conflict avoided to maintain congenial interactions. This interdependence is prominent in familialism with trust, respect, caring, duty, and supportive intergenerational relationships within the extended family. Ecuadorian parenting practices support this value ideology. It includes positive family processes, parental involvement, induction, and a democratic style that fosters belonging and connection to family that promotes social competence in children (Schvaneveldt, 2014). In a study of 63 countries, Chopik et al. (2017) found that Ecuador rated highest on empathy and social perspective taking, qualities that were associated with agreeableness, conscientiousness, emotional expression, and prosocial behaviours in collectivistic cultures.

Ecuador’s position on several other cultural dimensions in Hofstede’s model is interwoven with its collectivism. A high rating for power-distance indicates the acceptance of inequalities at all levels of society. This stance on inequities is typical of collective cultures (Komisarof & Akaliyski, 2025). Similarly, the country’s rating on short versus long-term orientation underscores its value for maintaining time-honoured traditions and norms along with a somewhat cautious perspective of change. In addition, Ecuador’s placement on Hofstede’s motivation toward achievement and success refers to the importance of hard work and a competitive orientation within one’s group to enhance its status and rewards. Taken together in the context of socio-economic changes, these cultural characteristics would be expected to influence the identity development of young people in Ecuador.

### The current study

This study investigated identity development using a person-centred analysis with the Dimensions of Identity Scales (DIDS; Luyckx et al., 2011) and the Identity Distress Scale (IDS; Berman et al., 2004, 2009) among university students in Ecuador. The empirically derived profiles were expected to approximate DIDS profiles generated in other countries (e.g. Luyckx et al., 2008a, 2008b; Schwartz et al., 2011; Skhirtladze et al., 2016). It was predicted that the profiles would be differentiated in terms of: (1) ego strengths (psychosocial maturity) and a standardised measure of identity status (diffusion, foreclosure, moratorium, achievement); and (2) in relation to academic, social, and personal-emotional adjustment at university; psychological symptoms; perceived global stress, agency, and optimism. More mature profiles (those high in commitment) were expected to be reflected in the standardised identity measures and to be associated with greater functional adjustment, mental health, and well-being.

## Method

### Participants

The sample included 418 Ecuadorian university students (mean_age_ = 20.8, SD = 3.1; median = 21 years; 66% female). Most identified as Mestizo/a (97.4%), with the others Indigenous (1%) and white (1.4%). In terms of marital status, 65.6% were single, 29.9% were married, and 4.5% divorced. The majority were living with parents (62.1%), other family members (10.8%), alone (9.4%), and the remainder with spouse, friends, or in residence.

### Measures

#### Predictors

The **Dimensions of Identity Development Scale** (DIDS; Luyckx et al., 2008a, 2008b) consists of 25-items with 5-items for each of the five scales: Commitment Making (CM) e.g. ‘I know what I want to do with my life’ (*α* = .87, *ω* = .88); Identification with Commitment (IC) e.g. **‘**My plans for the future offer me a sense of security’ (*α* = .86, *ω* = .89); Exploration in Breadth (EB) e.g. ‘I think a lot about the direction I want to take in my life’ (*α* = .62, *ω* = .63); Exploration in Depth (ED) e.g. ‘I think about the future plans I have made’ (*α* = .58, *ω* = .58); and Ruminative Exploration (RE) e.g. ‘I worry about what I want to do with my future’ (*α* = .79, *ω* = .78). Items are rated on a 5-point scale (1 = ‘strongly disagree’ to 5 = ‘strongly agree’) to indicate the extent to which they apply to the respondent. The DIDS has been validated and associated with adjustment in Belgium (Luyckx et al., 2008a, 2008b, 2013), Finland (Mannerstrom et al., 2017; Marttinen et al., 2016 ), France (Zimmermann et al., 2015), Georgia (Skhirtladze et al., 2016), Greece (Mastrotheodoros and Mott-Stefanidi, 2017), Hungary (Rivnyak et al., 2022), Japan (Hatano and Sugimura, 2017), Spain (Sanchez-Queija et al., 2024), and the United States (Johnson, 2012; Schwartz et al., 2011).

The **Identity Distress Scale** (IDS; Berman et al., 2004, 2009) measured interference or severe disturbance with identity development in terms of Identity Distress (DSM-III; APA, 1986) and Identity Problem (DSM-IV; APA, 1994). The IDS provides continuous measures for seven areas of difficulty (long-term goals, career choice, friendships, sexual orientation, religion, values and beliefs, and group loyalties). Items are rated on a 5-point Likert scale from 1 (‘not at all’) to 5 (‘very severely’) to indicate the extent to which respondents have been recently upset, distressed, or worried over each of these identity-related issues. Three additional items asked respondents to rate the overall level of discomfort these issues upset or distressed them, how much uncertainty these issues interfered with their life, and how long, if at all, they felt upset, distressed, or worried over these issues overall. This study used mean scores of the IDS-10 items (*α* = .80, *ω* = .79). The IDS has been validated in Bulgaria (Papazova & Bakracheva, 2017), Ecuador (Cordero-Hermida et al., 2024) and Poland (Janowicz et al., 2024) and used in studies with many countries including Belgium (Palmeroni et al., 2020), Spain, Canada (Gfellner & Córdoba, 2011; Gfellner & Cordoba, 2017; Gfellner & Cordoba, 2020), Italy (Sica et al., 2014), India, China, Taiwan, Japan (Berman et al., 2011; Caycedo et al., 2009), Sweden (Wangqvist & Frisen, 2011), and the United States (Berman et al., 2004, 2009, 2011; Berman & Montgomery, 2014).

#### Identity validation measures

The **Psychological Inventory of Ego Strengths** (PIES; Markstrom et al., 1997) measured maturity (ego strengths or values) in terms of Erikson’s eight stages. The 32-item short form consists of 4 items for each of the eight ego strengths (hope, will, purpose, competence, fidelity, love, care, and wisdom) that are rated on a 5-point Likert scale from 1 (‘does not describe me well’) to 5 (‘describes me very well’). Item scores are summed (*α* = .90, *ω* = .90). The PIES is validated in relation to standard measures of identity development, mental health, and functional well-being (Gfellner & Córdoba, 2011; Markstrom et al., 1997; Markstrom & Marshall, 2007).

The **Objective Measure of Ego Identity Status** (OMEIS-24; Adams, 1998) measured the four identity statuses: Foreclosure (*α* = .66, *ω* = .65), Achievement (*α* = .47, *ω* = .53), Moratorium (*α* = .62, *ω* = .59), and Diffusion (*α* = .47, *ω* = .46) as defined by Marcia, (1966). The short form consists of six items per scale, rated on a 6-point Likert scale from ‘strongly disagree’ to ‘strongly agree’ to indicate how each statement applies to oneself. Scores are summed for each of the identity statuses. Kroger & Marcia, (2011; Kroger & Marcia, 2022; Kroger et al., 2010) provide extensive reviews.

#### Outcome measures

The **Counselling Centre Assessment of Psychological Symptoms** (CCAPS-34; Locke et al., 2012), a screening instrument developed for use with university students, was the measure of psychological symptoms. The CCAPS-34 includes 34 items rated on a 5-point scale from 0 (‘not at all like me’) to 4 (‘extremely like me’) to indicate the extent to which the symptom has been experienced in the past two weeks. The scales include Depression (*α* = .86, *ω* = .85), Generalised Anxiety (*α* = .76, *ω* = .75), Social Anxiety (*α* = .78, *ω* = .78), Academic Difficulties (*α* = .72, *ω* = .75), Eating Concerns (*α* = .84, *ω* = .84), Hostility (*α* = .80, *ω* = .81), and Substance/Alcohol Use (*α* = .85, *ω* = .85). For each scale scores were tallied as well as a standardised Distress Index of scores on 20 items (CCAPS-DI; Youn et al., 2015; *α* = .92, *ω* = .91). Scale scores are consistent with established measures of psychological symptoms (Locke et al., 2012) and psychometric properties are given in the CCAPS User Manual (Centre for Collegiate Mental Health, 2015; Locke et al., 2012; McAleavey et al., 2012; Sherman et al., 2020). In a recent study Zhao et al. (2025) provided validation of the CCAPS for five ethnoraces in the United States.

**Perceived global stress** was indexed by ten items from standard stress scales that indicate students’ perceptions of general stress experienced over the past month (Gfellner & Córdoba, 2011). Items (e.g. ‘In the past month, how often have you thought that you could not cope with all the things you had to do?’) were rated on a 5-point scale from 1 (‘never’) to 5 (‘very often’) and summed for a composite stress score (*α* = .56, *ω* = .60).

**Optimism** was measured by the three positive items on the Life Orientation Test Revised Scale (LOT-R; Scheier et al., 1994). Items (e.g. ‘In uncertain times I usually expect the best.’) are rated on a 5-point scale from ‘strongly disagree’ to ‘strongly agree’ with a high score positive (*ω* = .26). Dispositional optimism is validated in relation to mental and physical health, coping, resilience, and quality of life (Ciro et al., 2010).

**Agency** was measured by the self-reliance scale from the four-factor model of emotional autonomy revised for use with EA (Lamborn & Groh, 2009). It assesses an internal-external orientation to adjustment with 7-items (e.g. ‘Luck decides most of the things that happen to me.’) rated on a 4-point scale from 1 = ‘strongly disagree’ to 4 = ‘strongly agree’ (*α* = .64, *ω* = .73).

The **Student Adjustment to College Questionnaire** (SACQ; Baker & Siryk, 1999) measured Academic (*α* = .78, *ω* = .77), Social (*α* = .85, *ω* = .84), and Personal-Emotional (PE; *α* = .81, *ω* = .81) functioning at university. The short form includes 27-items rated on a 9-point scale with end points labelled as ‘does not apply to me at all,’ to ‘’applies very closely to me’ to indicate the extent to which the statement applies to the respondent. The SACQ has been translated into many languages highlighting adaptations for cultural relevance (e.g. Donado et al., 2021 for Colombia) and psychometric robustness including internal consistency, test-retest reliability, construct, criterion, and predictive validity (Liu et al., 2025).

### Procedure

The instruments were translated by an Ecuadorian language teacher from Spanish to the ambient dialect of the region, and subsequently back translated to English by another Ecuadorian language teacher proficient in English. The survey items were then pilot tested with a sample of 10 Ecuadorian university students for understandability and relevance. Instructors in Psychology classes invited students to participate in the study and volunteers completed a survey under supervision in the university computer lab. Informed consent was required before students could access the survey and responses were confidential and surveys anonymized. Students were awarded a bonus point toward their final grade as a gratuity for participation.

Data was collected in 2018 in conjunction with the University of Cuenca project, ‘Calidad de Vida en Personas con Discapacidad y Variables Psicosociales Asociadas’ (Quality of life in people with disabilities and associated psychosocial variables). The project's University of Cuenca ethical approval code is: DIUC_XV_2017 005. The survey items for this study were adapted from a project in Canada. For this reason, the Brandon University Research Ethics approval is included (Certificate #200325).

### Data analysis

We first examined the data for outliers, and none were found. The exclusionary criterion eliminated participants with more than two missing items from the DIDS and the IDS to meet the MPlus analysis requirements. Latent profile analyses were then conducted in MPlus to generate a series of identity status profiles. We then examined possible group solutions from one to seven, using the best practices suggested by Spurk et al. (2020). Specifically, we compared solutions using relative fit criteria, entropy, and relevant likelihood ratios; these are presented in Table 1. We also examined possible solutions through the lens of available theory and previous research. The fit criteria suggested a five-group solution, while the likelihood ratios leaned towards a four-group solution. Looking at both, we felt that the five-group solution was more appropriate given both theoretical meaningfulness and the previous literature. When the appropriate groups were identified, a series of profile ANOVAs were run for all variables in the study.

**Table 1.** Latent profile analysis and fit indices for students in Ecuador profile membership percentages.

Profile	AIC	BIC	AdBIC	Entropy	Bootstrap LRT	LMR	1	2	3	4	5	6	7
1	5380.05	5428.31	5390.23	–	–		100%						
2	4879.98	4956.38	4896.09	.86	−2678.0*	502.13*	27%	73%					
3	4646.68	4751.23	4668.73	.83	−2429.9*	241.58	10%	45%	45%				
4	4436.06	4568.75	4464.04	.84	−2297.3*	291.42*	17%	10%	42%	31%			
5	4310.04	4470.88	4343.95	.86	−2185.0*	136.77*	21%	5%	11%	41%	22%		
6	4224.62	4413.61	4264.47	.85	−2115.0*	97.15	2%	13%	27%	18%	28%	11%	
7	4138.78	4355.92	4184.56	.86	−2065.3*	97.53	2%	18%	4%	25%	15%	25%	10%

Notes: * = *p* < .05.

## Results

**Figure 1.**
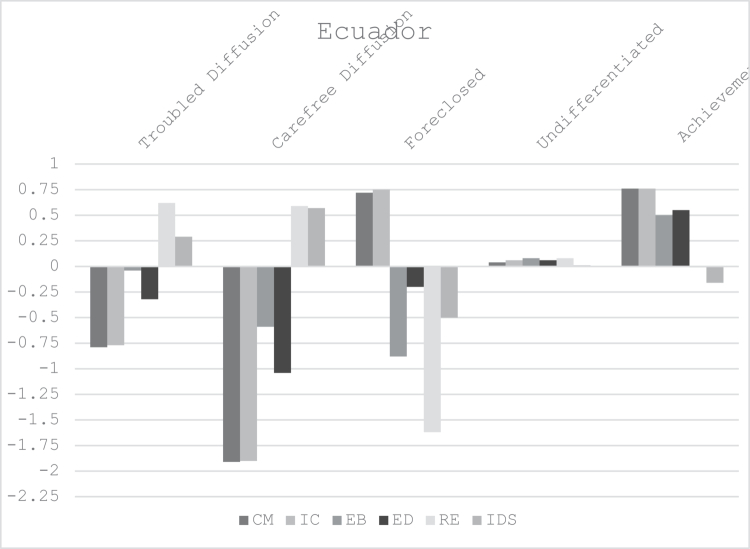
Latent Profiles of Students in Ecuador. Notes: CM = Commitment Making; IC = Identification with Commitment; EB = Exploration in Breath; ED = Exploration in Depth; RE = Ruminative Exploration; IDS = Identity Distress Scale.

Table 2 shows the five profiles generated for the DIDS and IDS scales. They included: Troubled Diffusion (20.4%), Carefree Diffusion (5.4%), Foreclosure (11.6%), Undifferentiated (40%), and Achievement (24.5%). These profiles align with the statuses reported in Europe (Luyckx et al., 2008a; Marttinen et al., 2016), North America (Gfellner et al., 2025; Johnson, 2012; Schwartz et al., 2011), and other countries (Hatano & Sugimura, 2017; Skhirtladze et al., 2016). Consistent with students in Belgium (Luyckx et al., 2008a, 2008b), Canada (Gfellner et al., 2025), and the USA (Johnson, 2012; Schwartz et al., 2011) Undifferentiated was the largest group. Figure 1 illustrates the identity dimensions relative to mean scores for the profiles.

**Table 2.** Summary of the person-centred profile ANOVAs of students in Ecuador.

Variable	Sample mean	Profile 1 troubled diffusion 20.4% (85)	Profile 2 carefree diffusion 5.4% (22)	Profile 3 foreclosed 11.6% (46)	Profile 4 undifferentiated 40% (169)	Profile 5 achieved 22.7% (90)	*F*-value	Eta
**DIDS** ^1^								
CM	3.98 (.78)	3.19 (.37)^c^	2.07 (.46)^d^	4.70 (.31)^a^	4.02 (.31)^b^	4.74 (.26)^a^	504.96***	.83
IC	3.98 (.78)	3.21 (.36)^c^	2.08 (.49)^d^	4.73 (.33)^a^	4.04 (.28)^b^	4.74 (.23)^a^	546.84***	.84
EB	3.89 (.65)	3.85 (.52)^b^	3.30 (.95)^c^	3.01 (.66)^d^	3.97 (.44)^b^	4.39 (.38)^a^	63.94***	.39
ED	3.68 (.63)	3.36 (.54)^c^	2.64 (.76)^d^	3.48 (.50)^b^	3.74 (.46)^b^	4.23 (.42)^a^	63.29***	.38
RE	3.22 (.93)	3.84 (.60)^a^	3.81 (.93)^a^	1.60 (.44)^c^	3.30 (.72)^b^	3.21 (.75)^b^	85.04***	.46
**Identity distress**	2.30 (.62)	2.59 (.64)^ab^	2.87 (.67)^a^	1.80 (.41)^d^	2.31 (.55)^bc^	2.14 (.56)^c^	21.66***	.18
AGE	20.5 (2.4)	20.6 (2.7) ^ab^	19.9 (1.5)^b^	21.7 (3.2)^a^	20.6 (2.3)^ab^	20.2 (2.3)^b^	3.47**	.03
**EOM-EIS-24** ^2^								
Diffusion	3.21 (.82)	3.61 (.80)^a^	3.74 (.83)^a^	2.87 (.65)^b^	3.15 (.79)^b^	2.98 (.80)^b^	12.27***	.11
Foreclosure	2.05 (.79)	2.02 (.75)	1.88 (.96)	1.76 (.71)	2.10 (.76)	2.15(.85)	2.34^NS^	.02
Moratorium	3.04 (.93)	3.48 (.91)^a^	3.56 (.97)^a^	2.38 (.70)^c^	3.10 (.89)^ab^	2.74 (.84)^bc^	17.02***	.14
Achievement	4.21 (.77)	3.92 (.61)^c^	3.34 (.68)^d^	4.29 (.86)^abc^	4.22 (.70)^abc^	4.59 (.74)^ab^	17.47***	.15
**Ego strengths**	3.63 (.58)	3.13 (.44)^c^	2.71 (.47)^d^	4.19 (.37)^a^	3.64 (.42)^b^	4.03 (.41)^a^	98.97***	.49
**SACQ**								
Academic	7.10 (1.2)	6.25 (1.0)^c^	5.64 (1.5)^d^	7.97 (.64)^a^	7.12 (.91)^b^	7.76 (.87)^a^	51.33***	.34
Social	6.01 (1.4)	5.22 (1.4)^c^	4.21 (1.5)^d^	6.52 (1.2)^ab^	6.01 (1.2)^b^	6.92 (1.1)^a^	35.21***	.26
Personal-emotional	4.6 (1.6)	3.84 (1.3)^c^	3.81 (1.3)^c^	5.75 (1.7)^a^	4.56 (1.6)^bc^	4.96 (1.6)^ab^	14.09***	.12
Global stress^3^	3.26 (.45)	3.29 (.45)^b^	3.51 (.32)^a^	3.02 (.46)^b^	3.23 (.46)^ab^	3.30 (.43)^ab^	2.10^ns^	.04
Optimism	3.6 (.57)	3.4 (.48)^bc^	3.3 (.51)^cd^	3.5 (.65)^ab^	3.6 (.51)^ab^	3.8 (.46)^a^	8.83***	.08
Agency	4.2 (.42)	3.9 (.41)^c^	3.8 (.20)^c^	4.5 (.30)^a^	4.2 (.39)^b^	4.3 (.36)^b^	26.39***	.21
CCAPS-DI^4^	1.35 (.79)	1.89 (.63)^a^	2.17 (.66)^a^	0.73 (.50)^c^	1.30 (.74)^b^	1.03 (.74)^bc^	35.27***	.26
**CCAPS scales**								
Depression	1.04 (.91)	1.60 (.81)^b^	2.05 (.99)^a^	0.39 (.45)^d^	1.03 (.85)^c^	0.62 (.76)^cd^	33.18***	.25
General anxiety	1.54 (.69)	1.98 (.79)^a^	2.02 (.84)^a^	0.99 (.72)^c^	1.51 (.85)^b^	1.37 (.95)^bc^	13.16***	.12
Social anxiety	1.64 (.94)	2.10 (.81)^b^	2.66 (.76)^a^	1.19 (1.0)^c^	1.60 (.86)^c^	1.28 (.84)^c^	21.29***	.17
Academic difficulties	1.27 (.87)	1.89 (.79)^b^	2.42 (.73)^a^	0.48 (.45)^d^	1.22 (.74)^c^	0.89 (.69)^c^	51.10***	.33
Eating problems	1.17 (1.2)	1.45 (1.3)^a^	1.32 (.1.2)^a^	0.88 (1.2)^a^	1.24 (1.1)^a^	0.87 (1.1)^a^	3.63**	.03
Hostility	1.26 (.89)	1.80 (.84)^a^	1.70 (.80)^a^	0.76 (.64)^b^	1.18 (.86)^b^	1.05 (.87)^b^	16.49***	.14
Alcohol/drug use	0.72 (.94)	0.99 (1.1)^a^	0.86 (.90)^a^	0.50 (.65)^a^	0.67 (.89)^a^	0.65 (.97)^a^	2.75*	.02

Notes: ^1^ = Dimensions of Identity Scales; ^2^ = Extended Objective Measure of Ego Identity Status; ^3^ = Student Adjustment to College Questionnaire; ^3^ = Global Stress (N = 199); ^4^ = CCAPS-24 Psychological Symptoms. ***p < .001; **p < .001; *p < .01; *p < .05; ^abc^ different superscripts indicate significant mean differences.

The profile ANOVAs are summarised in Table 2. Profile 1—Troubled Diffusion (20.4%) has scores below the mean for the commitment variables (CM, IC), at the mean on EB, and above the mean on RE and IDS. Profile 2—Carefree Diffusion (5.4%) indicates scores substantially below the mean on CM, IC, EB, ED, and above the mean on RE and IDS, respectively. Profile 3—Foreclosure (11.6%) reflects scores above the mean for the commitment variables and below the mean for all the exploration measures and identity distress. Profile 4—Undifferentiated (40%) shows scores near or at the mean for all scales. Profile 5—Achievement (24.5%) has scores above the mean for the commitment (CM, IC) and adaptive exploration scales (EB, ED), at the mean on RE, and below the mean on IDS.

`Age and sex differences are reported for comparison with other studies. As seen in Table 2, age attained significance with the oldest students in Foreclosure and the youngest in the Carefree Diffused and Achieved profiles. The other profiles (Undifferentiated and Troubled Diffused) did not differ from either of these extremes. The chi-square for sex by profile, ꭓ^2^​​ (4df) = 1.99, *p* = .74, was not significant. The profiles were then assessed for criterion validity in relation to Eriksonian psychosocial development indexed by ego strengths (Markstrom et al., 1997) and Adams (1998) OMEIS-24 measure of ego identity status. The results are given in Table 2. As expected, ego strength scores were significantly greater for students in the Achieved and Foreclosed than the Undifferentiated profiles, whose scores exceeded those of the Troubled Diffusion, and this profile scored significantly higher than the Carefree Diffused students. Although less dramatic, the same trend was evident for OMEIS-Achievement scores. Students’ scores in the Achieved profile did not differ from either the Foreclosed or Undifferentiated groups, and these later profiles aligned with the Troubled Diffused. As expected, Carefree Diffused students had the lowest OMEIS-Achievement scores.

Students in the Foreclosed profile had the lowest OMEIS-Diffusion scores but these did not differ from the Achieved and Undifferentiated profiles. As expected, the Carefree and Troubled Diffused groups indicated significantly lower OMEIS-Diffusion scores. Similarly, the lowest OMEIS-Moratorium scores were for students in the Foreclosed profile. Students in the Achieved profile aligned with the Foreclosed and the Undifferentiated groups, and the Undifferentiated students' OMEIS-Moratorium scores did not differ significantly from those in the diffused profiles.

The next set of analyses examined predictive validity of the profiles in relation to adaptive functioning at university, agency, optimism, perceived stress, and psychological symptoms. Table 2 provides the results of the profile ANOVAs for these variables.

As expected, Agency scores were elevated among Foreclosed students, followed by the Achieved and Undifferentiated (which did not differ), with the lowest scores for those in the Troubled and Carefree Diffused profiles (which did not differ). The highest Optimism scores were for the students in the Achieved profile and aligned with the Foreclosed and Undifferentiated groups. These later profiles differed from Optimism scores of students in the Carefree but not the Troubled Diffusion profile.

Academic adjustment reflected high commitment with elevated scores among the Foreclosed and Achieved profiles, followed by the Undifferentiated, and subsequently the Troubled and Carefree Diffusion groups, respectively. The trend was similar for Social Adjustment, with the highest scores for Achieved students in comparison with the Undifferentiated, and the Foreclosed did not differ from either of these groups. As expected, the lowest scores were for Carefree Diffusion students, followed by the Troubled Diffused group. The Foreclosed profile showed the greatest Personal-Emotional Adjustment; they aligned with students on the Achieved profile who did not differ significantly from the Undifferentiated students. Despite a score equivalent to the sample mean for the Undifferentiated group, this profile did not differ significantly from the Troubled and Carefree Diffused profiles with the lowest scores.

Similarly, as expected, the perceived Global Stress score was highest for Carefree Diffused students, followed by those in the Achieved profile. However, students in the Foreclosed profile did not differ from the Troubled Diffused, and those in the Achieved and Undifferentiated groups aligned with the former profiles. Nevertheless, students in the Foreclosed profile reported the lowest perceived stress score.

As predicted, psychological symptoms scores on the CCAPS-DI were greatest for students in the Diffused profiles and lowest for the Foreclosed. Students in the Foreclosed profile did not differ from Achieved students, and the Achieved students did not differ significantly from those in the Undifferentiated profile.

Similar trends were found for the psychological symptom scales with some noteworthy differences. Depression scores were highest for the Carefree Diffusion group, followed by the Troubled Diffused and then the Undifferentiated, which differed from the Foreclosed, and the Achieved did not differ from either of the former two groups. The same configuration of profile scores was seen for General Anxiety, but the Diffused groups did not differ. As with Depression, Social Anxiety scores were greatest among Carefree Diffused, followed by the Troubled Diffused students and subsequently, students in the Foreclosed, Achieved, and Undifferentiated profiles did not differ significantly from one another. Alternatively, Hostility scores significantly differed, with higher scores among the students in the diffused profiles in comparison with those in the other profiles, which did not differ from each other. Academic Difficulties scores were elevated among students in the Carefree Diffused profile, followed by the Troubled Diffused, and then the Differentiated and Achieved students who did not differ. As with Academic Adjustment on the SACQ, the lowest academic symptom scores were among students in the Foreclosed profile. Directional support was seen in the profile scores for Eating Problems and Alcohol/Drug Use although there were no significant mean-group differences despite significant effects.

## Discussion

The five profile solution is the model-based representation that aligned in general with those reported in person-centred analyses of the DIDS identity processes in Europe (Luyckx et al., 2008a, 2008b, 2013; Mannerstrom et al., 2017; Raemen et al., 2022; Vankerckhoven et al., 2023), the United States (Johnson, 2012; Schwartz et al., 2011), and other countries (Hatano & Sugimura, 2017; Skhirtladze et al., 2016). Similarly, in these studies differences in the identity profile configuration are related to culture and context.

Consistent with the identity framework, the Foreclosed and Achieved profiles had the highest and equivalent scores on the commitment variables. This reflects students’ acceptance at university in their chosen field of study. Education is a national priority in Ecuador (Guijarro-Garvi et al., 2022). At public universities, tuition is free contingent upon academic standing and requested-programme availability. It is a highly competitive system with little option of changing streams. Students make decisions on discipline and occupation when applying to university. These profiles accounted for slightly more than a third (34.3%) of the sample.

Apart from the Achieved, there was little evidence of exploration. A Moratorium profile was not found among these Ecuadorian students. Comparably, Luyckx et al. (2008a) reported Undifferentiated but no Moratorium clusters in samples of university freshmen and 12^th^ grade Belgian students. According to Marcia (1966) being uncommitted is the hallmark of classical moratorium, characterised by considerable anxiety and worry (as indicated in high RE) over a lack of productive exploration and no commitment (Luyckx et al., 2008a). Given the constraints of the tertiary educational system in Ecuador, this identity option may not be readily promoted within the college context or by traditional parenting styles (Schvaneveldt, 2014). In other words, experimenting and evaluating different academic areas and careers may not always be encouraged before making occupational choices.

The Undifferentiated was the largest profile (40%) with scores close to or at the sample means on each of the dimensions. It is consistent with what Adams (1998) labelled ‘low profile moratorium’ (Schwartz et al., 2011) used to categorise protocols that could not be clearly classified into any specific identity status. According to Adams et al. (2006) these individuals had a proclivity for identity development, but they reflect a reticent approach to proactive identity involvement. Such undifferentiated clusters accounted for the greatest proportion of university students in Belgium (Luyckx et al., 2008a), the US (Schwartz et al., 2011; Johnson, 2012), Canada (Gfellner et al., 2025), and Georgia (Skhirtladze et al., 2016). Similarly, in Ecuador, Undifferentiated students may be at a formative stage of identity work (Hatano & Sugimura, 2017; Johnson, 2012; Schwartz et al., 2011), and the university environment provides the venue to examine personal options before one is required to assume adult roles and social responsibilities (Arnett et al., 2014). In the global world, this aligns with a myriad of information sources available on the internet, social media internet, social media (e.g. Avci et al., 2025; Katzarska-Miller & Reysen, 2022; Kumar, 2025; Perez-Torres, 2024) as well as peer and scholastic interactions. However, Ecuadorian students are in designated fields of study with expectations for academic proficiency. Indeed, tracking over the college years is essential to assess the ways in which these undifferentiated students resolve identity issues.

For the diffusion profiles, the Carefree Diffused scored significantly below the Troubled Diffused (formerly called Diffused Diffusion) on the adaptive dimensions, indicating no consideration of identity work (Schwartz et al., 2011). Alternatively, the significantly higher scores for the adaptive exploration and commitment processes among the Troubled Diffusion students as well as consistency with the Undifferentiated group on EB, may be an early indicator of subsequent identity construction (Schwartz et al., 2011). Nevertheless, the similar low RE and identity distress scores of these diffused groups are unclear and may indicate differences in relation to potential identity unfolding. The high identity distress score for the Troubled Diffusion students is consistent with difficulties in resolving identity issues (Schwartz et al., 2011), yet for some students it may eventually function as a stimulus for exploration. In comparison, RE and identity distress may operate as an avoidance mechanism and maladaptive interference for Carefree Diffused individuals with no interest in identity concerns. As discussed later, the Carefree Diffused group may be at greater risk for serious psychological problems that impede identity development (Gfellner & Cordoba, 2020; Klimstra & Dennison, 2017). Follow-up of students in these diffused profiles is necessary to determine how identity work is resolved uniquely or whether Carefree Diffusion is a subset of troubled diffusion among these Ecuadorian students. This is essential to develop appropriate supportive services and intervention strategies.

As expected, the high commitment profiles evidenced advanced identity development in relation to ego strengths, psychosocial maturity in terms of Erikson’s eight stages (Markstrom et al., 1997). Similarly, the higher OMEIS-Achievement score for the Achieved profile reflects ongoing unobtrusive exploration not evidenced among foreclosed or undifferentiated students. Alternatively, the lowest OMEIS-Diffusion and moratorium scores for the diffused profiles indicate the general maladjustment seen in psychological symptoms and other areas. As with ego strengths and OMEIS-Achievement, Carefree Diffused students showed significantly greater disadvantage than Troubled Diffused students in academic and social adjustment at university, depression, social anxiety, and academic difficulties.

The predictions were supported for optimism, agency, and the college adjustment variables of students in the high commitment profiles. However, Foreclosed students demonstrated the highest academic proficiency as seen in academic agency, the lowest CCAPS academic difficulties score, and directionally for SACQ academic adjustment. Indeed, these qualities reflect academic resilience among students in collectivistic cultures associated with protective factors, including a competitive-based motivation to succeed, cooperation, school belonging, and a strong social connection to parents (Ozcan & Bulus, 2022). Similarly, Hofstede (2015) motivation toward achievement and success dimension underscores the importance of arduous work and a competitive orientation to enhance rewards and status within one’s family and collective. These characteristics are supported by familialism as seen in traditional Ecuadorian parenting practices (Schvaneveldt, 2014).

The trend was similar for psychological symptoms on the CCAPS-DI and most scales with clear differentiation between the Foreclosed, Achieved, and Differentiated in comparison with the diffused profiles. In terms of psychological difficulties, the Foreclosed and Achieved profile scores fell well below the sample means, the Undifferentiated placed at or near the sample means, and the Diffused scores exceeded the mean scores. On the CCAPS and symptom scales, the normal cutoff for clinical severity is 1.0 to indicate moderate and severe psychological symptoms and a need for intervention. In terms of mean scores, the greatest symptom severity was for the Carefree Diffused, somewhat less among the Troubled Diffused students, and considerably lower for the Undifferentiated. Although both the Foreclosed and Achieved profiles' scores fell within the normal range, scores were considerably lower among the Foreclosed students. In contrast to findings in other countries (Luyckx et al., 2008a, 2008b; Schwartz et al., 2011), these results indicate greater psychological adjustment among the students in Foreclosed than Achieved profiles. Similarly directional support was evident for personal-emotional adjustment at university.

Students in the Achieved profile reported the only adaptive exploration apart from the mean level of the Undifferentiated group. As with the Foreclosed, the Achieved students have clear identity goals, yet they remain open, flexible, and continue to evaluate current commitments. According to Carreno et al. (2024) identity complications for young people in developing South American countries may arise in relation to achieving a balance between their collectivistic norms and aspects of individualism associated Western societies. The reflective components of the adaptive exploration dimensions (Schwartz et al., 2011) as well as the positive stance of optimism may reflect this dissonance. In addition, an element of unease is indicated in the directionally higher mean scores on depression, general anxiety, CCAPS-DI, and the SACQ personal-emotional adjustment measures of the Achieved in comparison with the Foreclosed and closer approximation to the Undifferentiated profiles. The highest score on social adjustment at university suggests consideration of factors including other personal proclivities, characteristics, and aspects within the family, social, and university contexts that may relate to identity development among these Ecuadorian students.

In comparison with other countries, the elevated functional adaptation among Ecuadorian students in the Foreclosed profile mirrors the strong collectivistic culture (Chopik et al., 2017; Hofstede, 2015). Indeed, socio-economic changes in the context of traditional customs, norms, and values of group cohesiveness, belonging, familialism, and parenting styles influence identity development (Schvaneveldt, 2014; Torres Visuette et al., 2023) as reflected in the identity profiles. Further study is required to assess issues relevant to identity construction to ensure optimal mental health and well-being of young people in Ecuador.

There are several limitations of the study. First, the correlational design provides a static view of identity development that does not allow for the examination of prospective change over time. Further research requires longitudinal follow-up of students to document transitions in identity development and how they are related to functional outcomes. Second, the study relied on self-report measures. Further research would benefit from the use of qualitative measures that provide more nuanced information on relevant components of identity development. In addition, external sources of information from family, friends, academic records, and counselling services would provide objective material to supplement respondents' subjective reports. It is unclear how current findings would generalise to students attending private universities in Ecuador (Guijarro-Garvi et al., 2022) and to EAs who do not attend post-secondary institutions (Paz & Evans, 2023). Nevertheless, the latent profile analysis of Ecuadorian university students provides a comparative baseline from which to investigate trends in the progress of identity construction and factors that influence its development.

## Data Availability

The data for the study are available on reasonable request from the corresponding author.
